# Draft Genome Sequence of the Polyextremophilic *Halorubrum* sp. Strain AJ67, Isolated from Hyperarsenic Lakes in the Argentinian Puna

**DOI:** 10.1128/genomeA.01096-13

**Published:** 2014-02-06

**Authors:** Germán F. Burguener, Marcos J. Maldonado, Santiago Revale, Darío Fernández Do Porto, Nicolás Rascován, Martín Vázquez, María Eugenia Farías, Marcelo A. Marti, Adrián Gustavo Turjanski

**Affiliations:** a Plataforma de Bioinformática Argentina, Instituto de Cálculo, Pabellón 2, Ciudad Universitaria, Facultad de Ciencias Exactas y Naturales, UBA, Buenos Aires, Argentina; b Laboratorio de Investigaciones Microbiológicas de Lagunas Andinas (LIMLA), Planta Piloto de Procesos Industriales y Microbiológicos (PROIMI), CCT-CONICET, Tucumán, Argentina; c Cátedra Microbiología Agrícola, Facultad de Ciencias Agrarias, Universidad Nacional de Jujuy, San Salvador de Jujuy, Argentina; d Instituto de Agrobiotecnología de Rosario (INDEAR), CONICET, Predio CCT, Rosario, Argentina; e Departamento de Química Biológica, Pabellón 2, Ciudad Universitaria, Facultad de Ciencias Exactas y Naturales, UBA, INQUIMAE/CONICET, Buenos Aires, Argentina

## Abstract

*Halorubrum* sp. strain AJ67, an extreme halophilic UV-resistant archaeon, was isolated from Laguna Antofalla in the Argentinian Puna. The draft genome sequence suggests the presence of potent enzyme candidates that are essential for survival under multiple environmental extreme conditions, such as high UV radiation, elevated salinity, and the presence of critical arsenic concentrations.

## GENOME ANNOUNCEMENT

The high-altitude Andean Lakes (HAAL) consist of several shallow lakes located in a desert known as Puna, located at >3,000 m altitude. They are exposed to extreme environmental conditions, such as high UV radiation levels, elevated salinity, and heavy metals and metalloids, mainly arsenic (1–5). Interestingly, *Halorubrum* was identified as the dominant haloarchaeal taxon in HAAL.

The extremely halophilic archaeon *Halorubrum* sp. strain AJ67 was isolated from Laguna Antofalla located in Catamarca, Argentina, in the Argentinian Puna. The 16S rRNA gene sequence shows a relationship to species of the genus *Halorubrum*, with a close relationship to *Halorubrum chaoviator* (98.74% identity). AJ67 belongs to a single euryarchaeotal order (*Halobacteriales*) that inhabits hypersaline environments (3 to 5 M), such as salt lakes, salt ponds, and marine salterns. The genus *Halorubrum* belongs to the family *Halobacteriaceae* (6). Previous molecular ecological studies showed that archaeal halophiles dominate these ecosystems (7–9) and that the genus *Halorubrum* (6, 10) is widely distributed in hypersalinic habitats (11–13). The cells are Gram-negative, rod shaped, and motile. The colonies are small, red-orange pigmented, and smooth. AJ67 is a chemo-organotrophic and aerobic archaeon.

The genome sequence was obtained using a whole-genome shotgun (WGS) strategy with a 454 GS Titanium pyrosequencer at the Instituto de Agrobiotecnología Rosario (INDEAR), Argentina. Assembly was done using 454 Newbler version 2.6, using the -urt option, with 8.25× genome coverage. This assembly generated 50 scaffolds. The draft genome is 4,225,006 bases in length. The G+C content of the genomic DNA is 63.71 mol%. Genome annotation was done using the standard operating procedures (SOPs) from the Integrative Services for Genomic Analysis (ISGA) (14) and our own prokaryotic annotation pipeline. The RAST annotation server was also used for subsystem descriptions (15). A total of 3,370 coding sequences (CDSs) and 49 structural RNAs (43 tRNAs) were predicted. A total of 1,086 CDSs (32%) were classified as hypothetical proteins. According to RAST, the annotation identified 489 CDSs (14%) into RAST subsystems. The genome of *Halorubrum* sp. AJ67 presents 21 genes devoted to resistance to toxic compounds, such as antibiotics, arsenic, cadmium, mercury, and others, according to RAST (15). The high resistance to arsenic previously observed in AJ67 can be explained based on the greater number of genes encoding the detoxification of this compound, 17 genes in comparison to 15 genes observed in *Haloquadratum walsbyi* DSM 16790 and in *Natronomonas pharaonis* DSM 2160. A striking difference with other *Halobacteriaceae* genomes is the presence of the arsenite oxidase (*aio*) gene, which oxidizes arsenite and reduces oxygen or nitrate, arsenite being one of the most toxic arsenic species. This is the first report of the presence of the *aio* gene in this genus. Strain AJ67 contains a complete DNA repair system, including UvrABC (subunit A and B) and photolyase, which confers high resistance to UV radiation.

### Nucleotide sequence accession number.

This whole-genome shotgun project has been deposited at DDBJ/EMBL/GenBank under the accession no. CBVY000000000. The version described in this paper is the first version.
